# Comparison of clinical safety and feasibility between reduced-port laparoscopic radical gastrectomy and conventional laparoscopic radical gastrectomy: A retrospective study

**DOI:** 10.3389/fsurg.2022.995194

**Published:** 2022-09-28

**Authors:** Liang Wang, Yingfang Deng, Su Yan, Xinfu Ma, Cheng Wang, Wei Miao, Xiaoqian Chen

**Affiliations:** ^1^ Gastrointestinal Oncology Surgery, Affiliated Hospital of Qinghai University & Affiliated Cancer Hospital of Qinghai University, Xining, China; ^2^ Medical-Oncology, Affiliated Hospital of Qinghai University & Affiliated Cancer Hospital of Qinghai University, Xining, China

**Keywords:** conventional laparoscopic surgery, reduced port laparoscopic surgery, single-incision laparoscopic surgery, natural orifice specimen extraction surgery, gastric cancer

## Abstract

**Background:**

Traditional open gastric cancer surgery has evolved from porous to reduced-hole, single-hole, or even natural cavity surgery to laparoscopic surgery, due to the continuous development of minimally invasive concepts and medical technologies, as well as awareness for the concept of rapid recovery. Conventional laparoscopic radical gastrectomy is quite mature in age at the moment, but how to progress to minimally invasive surgery without increasing the difficulty of surgery while ensuring clinical safety and feasibility is worth further investigation. Therefore, the clinical safety and feasibility of reduced port laparoscopic radical gastrectomy were assessed in this study.

**Methods:**

Information on the clinical data of patients undergoing laparoscopic radical gastric cancer surgery in a single centre between May 2020 and May 2022 was collected, and a total of 232 patients were included in this study according to the study protocol design. The clinical data of 232 patients with gastric cancer treated by two different surgical methods, namely, reduced port laparoscopic surgery (RPLS) or conventional laparoscopic surgery (CLS), were retrospectively analysed. The intraoperative indices, postoperative pathological indices, and short-term postoperative complications (within 30 days) of the two different surgical methods were evaluated, as well as the surgical methods’ feasibility and short-term postoperative recovery effect.

**Results:**

There was no significant difference between the general data of patients with RPLS and CLS (*P >* 0.05). Compared with CLSG, the operation time, digestive tract reconstruction time and lymph node dissection time of RPLSG are shorter. The intraoperative blood loss was less, and the incision was minimally invasive (*P* *<* 0.05). In the short-term postoperative effect, the level of white blood cell count on the first day, the time of getting out of bed, the time of removing drainage tube, the time of hospitalization and the VAS of pain on the first, third and fifth days after operation, RPLSG was obviously superior to CLSG (*P* *<* 0.05). There was no significant difference between RPLSG and CLSG in terms of pathological indices (*P* *>* 0.05).

**Conclusions:**

The treatment of gastric cancer with RPLS has good safety, feasibility and short-term postoperative effects, which is in line with the implementation of the modern concept of rapid rehabilitation surgery.

## Introduction

The laparoscopic technique has been gradually utilized in the surgical treatment of early gastric cancer since the application of laparoscopic-assisted radical resection of regional gastric cancer was first reported in 1994 by Kitano et al. (1). Research results of JLSSG-0901 (2) in Japan, KLASS-02 (3) in South Korea and Class-01 (4) in China indicated that laparoscopic radical gastrectomy for locally advanced gastric cancer by professional surgeons did not increase major surgical complications (5, 6). Laparoscopic magnification technology not only enables viewing of fine structures in the vascular system, nerve and fascia in detail, but with the development of endoscopic technology, this further allows the operator to have a special advantage in the clear identification of each anatomical level during the operation. Compared with traditional open surgery, laparoscopic surgery is associated with less pain, less blood loss, a more beautiful incision, fewer inflammatory reactions, faster recovery of gastrointestinal function and shorter hospital stays (7). A consensus, it is widely used in surgical treatment. Conventional laparoscopic surgery (CLS) is mostly conducted by the 5-port method. However, single-incision laparoscopic surgery (8) (SILS) is a single incision of approximately 4 cm (9) through the natural folds of the umbilical region that is placed in a single-port operating platform. The operation is completed through multiple channels on the platform, and it is mostly used for gallbladder and appendix operations (10, 11). Reduced port laparoscopic surgery (RPLS), on the other hand, is based on a single incision through the navel, similar to SILS, and a 12 mm trocar hole is added to the left upper abdomen, through which the abdominal drainage tube can be placed after surgery. The clinical data of 232 gastric cancer patients who met the research plan were retrospectively compared in this study, and the clinical safety and feasibility of laparoscopic radical gastrectomy with a reduced port were assessed.

## Materials and methods

### Patients

Information on the clinical data of patients undergoing laparoscopic radical gastric cancer surgery in a single centre between May 2020 and May 2022 was collected, and a total of 232 patients were included in this study according to the study protocol design, with 176 male patients and 56 female patients and an average age of 57.57 ± 10.04 years. They were divided into two groups: CLS (*n* = 116) and RPLS (*n* = 116). The Ethics Committee of Qinghai University's Affiliated Hospital approved the study (approval letter ethics batch number: *P*-SL-20190003), and the patients and their families signed an informed consent form. All of the operations were performed by the same surgical team.

### Inclusion and exclusion criteria

Inclusion criteria were: (1) Age 18–80 years; (2) Before operation, diagnosis was confirmed by pathological biopsy with an ultrasonic gastroscope, and the location and clinical stage of the lesion were further confirmed by contrast-enhanced CT examination of the stomach; (3) Preoperative imaging examination excluded distant metastasis to the liver, lung and other organs; (4) The pathological diagnosis after laparoscopic radical gastrectomy was R0 resection; and (5) Complete clinical data.

Exclusion criteria were: (1) Stage T4b tumour, preoperative existence of fusion lymph nodes, or distant metastasis of tumour; (2) Emergency surgical treatment for complications such as gastric bleeding and perforation before operation; (3) Palliative treatment or conversion to laparotomy during operation; (4) Neoadjuvant chemotherapy before operation; and (5) Incomplete clinical data.

### Operation method and postoperative treatment

**Figure 1:**
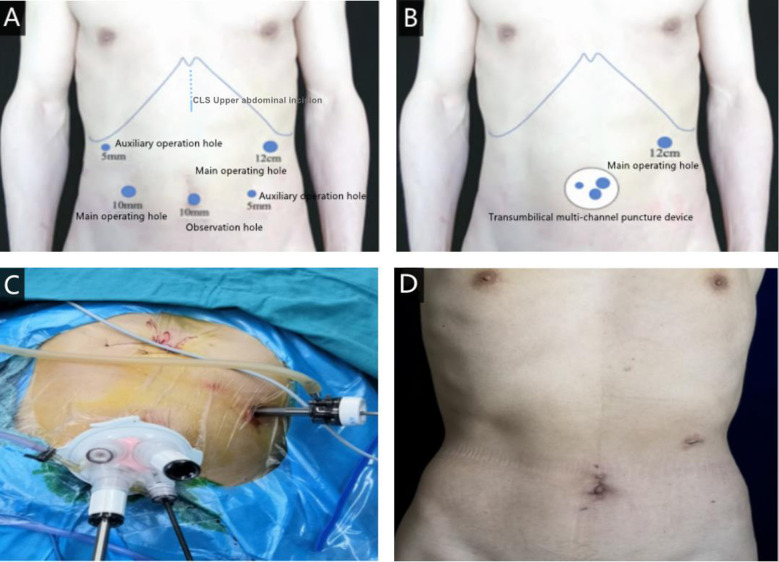
(**A**) the layout of the surgical puncture port of CLS; (**B**) the layout of the surgical puncture port of RPLS; (**C**) intraoperative incision position of RPLS; (**D**) postoperative abdominal wall incision after RPLS.

The operation methods and postoperative treatment measures were explained to the patients in detail before the operation. According to the patients' wishes, the CLSG (conventional laparoscopic surgery group) or the RPLSG (reduced port laparoscopic surgery group) was freely chosen, and the consent form was signed for the selected operation. The scope of gastric resection and lymph node dissection were all implemented according to the provisions of the «Fifth Edition of Japanese Gastric Cancer Treatment Guidelines» (10). The CLS is laid out in the conventional five-port method, with a 1-cm-long arcing port along the inferior border of the umbilicus. A 12 mm trocar and a 5 mm trocar were placed 2 cm below the intersection of the anterior axillary line and the rib arch on each side. A 10 mm trocar and a 5 mm trocar were placed at the intersection of the horizontal Line 2 cm above the umbilicus and the lateral border of the rectus abdominis muscle. For RPLS, a 3–5 cm long curved incision was made around the umbilicus at the natural fold of the umbilicus, and a single-port operating platform was placed into the abdominal cavity layer by layer. A 12-mm trocar was then placed 2 cm below the intersection of the patient's left midclavicular line and rib margin. The layout of the surgical puncture port in both groups is shown in Figures 1A,B. The patient was placed in a supine split-legged position intraoperatively, as shown in Figure 1C. The postoperative abdominal wall incision of the RPLSG patient is shown in Figure 1D. For CLS operator position: The main knife is located on the left side or between the legs of the patient, the first aid is located on the right side of the patient, and the laparoscopic assistant is located between the legs or on the right side of the patient. For RPLS operator's position: The main knife is located between the legs of the patient, and the laparoscopic assistant stands on the right side of the patient.

### Observation index

**General information:** sex, age, body mass index, American Society of Anaesthesiologists (ASA) grade, previous abdominal surgery history, tumour length and diameter, tumour location, and tumour differentiation degree;
**Intraoperative indicators:** operation time, digestive tract reconstruction time, lymph node dissection time, intraoperative blood loss, and total length of abdominal incision;
**Postoperative pathological indices:** the total number of lymph nodes, the positive number of metastatic lymph nodes, the distance of the oral margin, the distance of the anal margin, and pT stage, pN stage and pTNM stage.
**Postoperative recovery:** laboratory test indices, postoperative time to getting out of bed, postoperative exhaust time, postoperative intake of liquid diet time, drainage tube removal time, postoperative hospitalization time, visual analogue scale (VAS) on the 1st, 3rd and 5th postoperative days;
**Postoperative safety indicators:** Complications include anastomotic leakage, anastomotic bleeding, pulmonary infection, incision-related complications and pancreatic fistula (Clavien‒Dindo Grades II and III) (12).

### Statistical analysis

SPSS 25.0 statistical software was used to analyse the data. When the measurement data were in accordance with the normal distribution, the *t* or t^/^ test of two independent samples was used and expressed by *(X ± S)*; when it did not conform to the normal distribution, the rank sum test was used and expressed by *M (Q_L_−Q_U_)*. The qualitative data were tested by the *X*^2^ test. When *P* < 0.05, the difference was considered statistically significant. GraphPad Prism 7.00 software was used for statistical graphs.

## Results

### Preoperative general information

According to the research plan, 232 patients with gastric cancer were included in this study, with 116 patients in the CLSG, including 86 males (74.13%) and 36 females (25.87%), with an average age of 56.76 ± 9.37 years old. In addition, 116 patients were in the RPLSG, including 90 males (77.58%) and 26 females (22.42%). The average age for the RPLSG was 58.39 ± 10.65 years old. Statistical analysis showed that there was no significant difference in sex ratio or age between the two groups. Moreover, there was no significant difference between the two groups in BMI (body mass index), ASA (American Society of Anaesthesiologists score) grade, history of previous abdominal surgery, tumour major axes, tumour minor axes, tumour location, or degree of differentiation (see Table 1).

**Table 1:** Preoperative patient demographic information.

	CLSG (n = 116)	RPLSG (n = 116)	*P*-value
Age (years)	56.76 ± 9.37	58.39 ± 10.65	0.220
Gender (%)			
Male	86 (74.13%)	90 (77.58%)	0.539
Female	30 (25.87%)	26 (22.42%)	
BMI (kg/m^2^)	23.11 (20.20–25.58)	22.06 (20.00–24.42)	0.067
ASA grade (%)			
I	4 (3.44%)	9 (7.75%)	0.194
II	85 (73.27%)	74(63.79%)	
III	27 (23.29%)	33 (28.46%)	
History of previous abdominal surgery (%)			
No	70 (60.34%)	83 (71.55%)	0.096
Yes	46 (39.66%)	33 (28.45%)	
Tumour major axes (cm)	3.50 (3.00–4.00)	3.00 (2.52–3.90)	0.066
Tumour minor axes (cm)	3.00 (2.00–3.00)	2.40 (1.90–3.00)	0.073
Tumour location (%)			
Upper 1/3 of the stomach	36 (31.03%)	32 (27.58%)	0.221
Middle 1/3 of the stomach	28 (24.13%)	40(34.48%)	
Lower 1/3 of the stomach	52 (44.84%)	44 (37.94%)	
Degree of differentiation (%)			
Highly differentiated	10 (8.62%)	5 (4.31%)	0.270
Intermediate differentiation	32 (27.58%)	40 (34.48%)	
Low differentiation	74 (63.80%)	71 (61.21%)	

### The time of RPLS is shorter, the amount of blood loss is less, and the incision is less invasive

**Figure 2:**
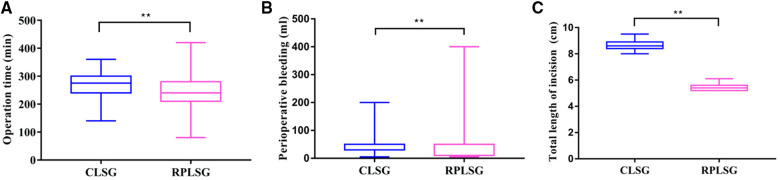
(**A**) operation time of different surgical methods; (**B**) effect of different surgical methods on perioperative bleeding; (**C**) effect of different surgical methods on total length of incision.

No patients were converted to laparotomy after undergoing laparoscopic radical gastrectomy for gastric cancer in either group. The operation was completed successfully by all 116 RPLS patients, with no additional puncture holes required. The comparison of intraoperative indices between the two groups showed that RPLSG was shorter than CLSG in operation time (Figure 2A), digestive tract reconstruction time and lymph node dissection time (*P* < 0.05). Compared with CLSG in intraoperative blood loss and total length of abdominal incision (all trocar puncture sites and auxiliary incisions are included), RPLS was significantly more minimally invasive (*P* < 0.05) (Figures 2B,C) (see Table 2).

**Table 2:** Intraoperative correlation index.

	CLSG (*n* = 116)	RPLSG (*n* = 116)	*P*-value
Operation Time (min)	275 (240–300)	240 (210–280)	0.002
Digestive tract reconstruction time (min)	80 (70–100)	70 (60–80)	0.001
Lymph node dissection time (min)	200 (170–220)	170 (140–190)	0.001
Intraoperative bleeding volume (ml)	50 (30–50)	10 (10–50)	0.001
Total length of abdominal incision (cm)	8.60 (8.40–8.90)	5.40 (5.20–5.60)	0.001

### RPLS can achieve the same radical effect as CLS

In terms of postoperative pathological indices of the two groups, we found that there was no statistical significance in the total number of lymph nodes obtained, positive number of metastatic lymph nodes, distance of oral margin, distance of anal margin, pT stage, pN stage or pTNM stage (*P > *0.05) (See Table 3).

**Table 3:** Postoperative pathological indices.

	CLSG (*n* = 116)	RPLSG (*n* = 116)	*P*-value
Total number of lymph nodes obtained	34.43 ± 15.07	35.06 ± 13.03	0.734
Number of positive lymph node metastases	1.00 (0.00–7.00)	1.00 (0.00–7.00)	0.949
Mouth-side margin distance (cm)	2.50 (1.50–4.00)	2.50 (1.00–4.50)	0.394
Anal margin distance (cm)	3.75 (2.00–6.00)	3.65 (2.00–6.00)	0.728
Staging of pT (%)			
pT_1_ stage	16 (13.79%)	20 (17.24%)	0.443
PT_2_ stage	26 (22.41%)	23 (19.82%)	
PT_3_ stage	51 (43.96%)	42 (36.20%)	
PT_4_ stage	23 (19.84%)	31 (26.74%)	
Staging of pN (%)			
pN_0_ stage	53 (45.68%)	50 (43.10%)	0.172
pN_1_ stage	13 (11.20%)	25 (21.55%)	
pN_2_ stage	17 (14.65%)	12 (10.34%)	
pN_3_ stage	33 (28.47%)	29 (25.01%)	
Staging of pTNM (%)			
I stage	33 (28.44%)	28 (24.13%)	0.684
II stage	32 (27.58%)	31 (26.72%)	
III stage	51 (43.98%)	57 (49.15%)	

### RPLS can reduce postoperative inflammatory reactions and pain and can accelerate the postoperative recovery of patients

**Figure 3:**
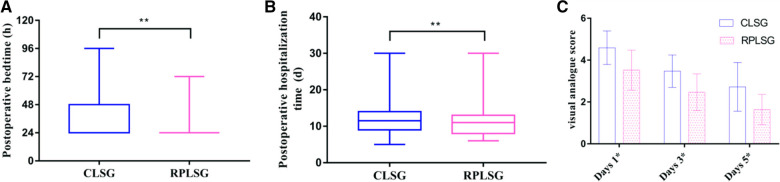
(**A**) effect of different surgical methods on postoperative bedtime; (**B**) effect of different surgical methods on postoperative hospitalization time; (**C**) the effect of different surgical methods on postoperative pain on days 1, 3, and 5.

In terms of postoperative recovery, there were statistically significant differences between the two groups in the levels of white blood cell count measured on the first day, albumin measured on the third day, postoperative bed time, postoperative exhaust time, postoperative feeding time, drainage tube removal time, postoperative hospitalization time and VAS score at one day, three days, and five days after operation (*P < *0.05) (Figures 3A–C). However, there was no significant difference in white blood cell count, haemoglobin or total bilirubin on the third and fifth days (*P* *>* 0.05) (See Tables 4, 5).

**Table 4:** Postoperative recovery index.

	CLSG (*n* = 116)	RPLSG (*n* = 116)	*P*-value
Postoperative bedtime (h)	48.00 (24.00–48.00)	24.00 (24.00–24.00)	0.001
Postoperative time to exhaustion (h)	72.00 (48.00–72.00)	48.00 (48.00–72.00)	0.001
Postoperative feeding time (d)	9.00 (7.00–10.00)	8.00 (6.00–9.00)	0.002
Drainage tube removal time (d)	10.00 (8.00–12.00)	8.00 (5.00–11.00)	0.001
Postoperative hospitalization time (d)	11.50 (9.00–14.00)	11.00 (8.00–13.00)	0.007
VAS
Day 1	4.59 ± 0.80	3.52 ± 0.95	0.001
Day 3	3.45 ± 0.77	2.46 ± 0.87	0.001
Day 5	2.72 ± 1.16	1.63 ± 0.72	0.001

**Table 5:** Postoperative recovery index.

	CLSG (*n* = 116)	RPLSG (*n* = 116)	*P*-value
White blood cell count (10^9^/L)			
Day 1	11.39 (9.86–14.16)	10.67 (8.79–12.60)	0.014
Day 3	8.37 (6.12–10.40)	7.89 (6.07–9.90)	0.291
Day 5	6.13 (4.92–8.25)	6.04 (5.01–7.87)	0.841
Albumin level (g/L)			
Day 1	35.60 (33.30–38.37)	36.70 (33.82–38.80)	0.062
Day 3	36.55 (34.05–38.07)	36.95 (35.00–39.72)	0.005
Day 5	36.90 (34.55–40.15)	38.00 (35.62–40.57)	0.112
Haemoglobin level (g/L)			
Day 1	129.96 ± 26.33	127.38 ± 24.65	0.442
Day 3	115.07 ± 21.68	116.43 ± 21.55	0.634
Day 5	113.32±21.35	114.26±21.53	0.739
Total bilirubin level (μmol/L)			
Day 1	13.80 (9.52–19.62)	14.70 (9.82–22.80)	0.365
Day 3	17.80 (14.00–25.07)	18.10 (13.12–25.57)	0.900
Day 5	20.10 (14.17–29.30)	17.85 (14.25–29.30)	0.472

### RPLS has the same security as CLS and can reduce the occurrence of SSI

**Figure 4:**
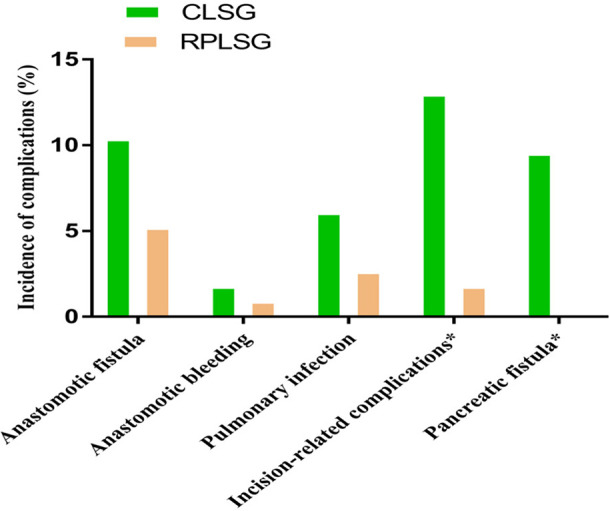
Effect of different surgical methods on the incidence of postoperative complications.

In terms of postoperative safety indicators, there was no significant difference in the incidence of anastomotic leakage, anastomotic bleeding or pulmonary infection between the two groups (*P >* 0.05), but there were significant differences in the incidence of incision-related complications and pancreatic fistula (*P <* 0.05) (Figure 4) (See Table 6).

**Table 6:** Postoperative safety index.

	CLSG (*n* = 116)	RPLSG (*n* = 116)	*P*-value
Anastomotic fistula (%)			
No	104 (89.65%)	110 (94.82%)	0.219
Yes	12 (10.35%)	6 (5.18%)	
Anastomotic bleeding (%)			
No	114 (98.27%)	115 (99.13%)	1.000
Yes	2 (1.73%)	1 (0.87%)	
Pulmonary infection (%)			
No	109 (93.96%)	113 (97.41%)	0.333
Yes	17 (6.04%)	3 (2.59%)	
Incision-related complications (%)			
No	101 (87.06%)	114 (98.27%)	0.002
Yes	15 (12.94%)	2 (1.73%)	
Pancreatic fistula (%)			
No	105 (90.51%)	116 (100%)	0.001
Yes	11 (9.49%)	0 (0.00%)	

## Discussion

With the increasing development of minimally invasive and standardized surgery, laparoscopic surgery has evolved from multiport to reduced-port, single-port, and even natural orifice specimen extraction surgery (NOSES) (13). The progression of minimally invasive surgery technology is the result of the combined advancement of surgical concepts, surgical instruments, and surgical techniques. The aim of minimally invasive surgery is to provide a painless and scar-less surgical approach (14). In terms of cosmetology and accelerated rehabilitation surgery, a large number of literature reports (15, 16) show that laparoscopic radical gastrectomy is superior to open surgery. Traditional laparoscopic radical gastrectomy for gastric cancer uses a five-port method with or without liver suspension, and gastric dissociation, lymph node dissection, and digestive tract reconstruction are completed with the help of assistants. Omori et al. (15) were the first to report the use of single-port laparoscopic radical gastrectomy for early distal gastric cancer in 2011. However, because of the lack of triangular positioning between the surgical instruments and the abdominal lens, coaxial effects easily occur, which limits the operating range of the surgical area and causes rear-end collisions between surgical instruments—not only increasing the operation difficulty but also placing higher demands on the supporting surgical team ^(^17). Simultaneously, more clinical trials are required to confirm the curative effect of oncology, lymph node dissection, and digestive tract reconstruction. As a result, single-port laparoscopic radical gastrectomy development is limited, and it is more frequently used in simple operations such as cholecystectomy and appendectomy (18–21). In contrast, RPLS uses an additional 12-mm poking port 2 cm below the intersection of the left midclavicular line and the rib margin as the main operating port of the main knife minus the two holes of the assistant, based on the SILS. This method can facilitate the clearance of regional lymph nodes in the suprapancreatic region and the splenic hilar region, while overcoming the operational drawbacks associated with single-port laparoscopy. At the same time, it can be used to place the abdominal drainage tube without making another abdominal incision, which reduces damage to the abdominal wall blood vessels and nerves, not only improving surgical safety but also balancing the relationship between surgical safety and minimally invasive surgery. As a result, some surgeons will attempt to use RPLS with laparoscopic assistance to complete gastric dissociation, lymph node dissection, and digestive tract reconstruction.

Although CLS is less difficult than RPLS and should take less time in operation, digestive tract reconstruction, and lymph node dissection, the results of this study show that RPLS takes less time in operation, digestive tract reconstruction, and lymph node dissection, contradicting conventional knowledge. The absence of the trocar incision in RPLS may lead to an increase in the difficulty of the procedure and a prolongation in time of the procedure. When an RPLS surgeon has completed the RPLS learning curve and their surgical technique and proficiency have improved, the precision of intraoperative operations will be increased. At the same time, the surgeon can complete gastric dissociation, lymph node dissection, and digestive tract reconstruction with the help of a laparoscopic assistant, and the coordination of one person's actions is better than that of the assistant's, which is one of the main reasons for shortening the operation time, digestive tract reconstruction time, and lymph node dissection time. Furthermore, in conventional laparoscopic radical gastrectomy for gastric cancer, the assistant frequently causes tissue traction and accessory damage to organs in the operation area due to insufficient cooperation, resulting in a corresponding extension of the operation procedure and an increase in postoperative complications (22–23). RPLS, on the other hand, is operated independently by the chief surgeon, which can avoid issues caused by improper operation team cooperation, thus improving operation efficiency and reducing intraoperative blood loss. The CLS multiple trocar puncture port approach may decrease patient satisfaction with the postoperative aesthetics of the abdominal wall incision. In addition, this approach also increases the risk of complications associated with trocar port herniation, infection, and metastatic tumour cell implantation. After the completion of endoscopic dissociation, CLS requires reselection of the abdominal wall incision to remove the specimen. However, the reselection of the incision will inevitably lead to a longer operative time. It also increases the total length of the abdominal incision because of the increased number of trocar puncture ports, which may lead to an increased incidence of intraoperative and postoperative abdominal infection and surgical-site infection (SSI) (24). This results in increased postoperative pain, delayed incisional healing, reduced abdominal wall aesthetics, and increased financial costs and psychological burden for the patient. In contrast, for RPLS, there is no need to reselect the abdominal wall incision, and the operation is completed by removing the specimen through a single incision in the umbilicus, using the curvature of the umbilicus after pulling out the single-port operating platform. This brings great convenience to the operation. The umbilical incision has natural folds due to the low fatty and muscle tissue content in the abdominal wall layer. Postoperatively, the incision is better concealed than CLS, and the patient has better postoperative abdominal wall aesthetics with less postoperative pain. This also facilitates early postoperative bed and out-of-bed activities and promotes rapid recovery of patient function. As RPLS is less invasive, it can reduce the postoperative inflammatory response and has greater advantages in accelerating postoperative rehabilitation in patients under the condition of a single operation to avoid side injury and shorter operation time. Simultaneously, under the condition of a single operation, the amount of postoperative exudation is reduced to avoid side injury and shorter operation time, and the time of abdominal cavity extubation is shortened, avoiding the delay of extubation—which is usually caused by an increase in exudation and increases the probability of abdominal cavity and incision infections and the economic and psychological burden on patients. In terms of postoperative safety and pathological indicators, there was no significant difference in the incidence of anastomotic leakage, anastomotic bleeding, pulmonary infection, or the total number of lymph nodes between the two groups, indicating that RPLS can still achieve the same radical effect as CLS without increasing postoperative complications, but the use of reduced-port laparoscopy in radical treatment of gastric cancer still requires a large number of clinical studies for further confirmation.

RPLS technology was developed on the basis of CLS, which avoided the difficulty of SILS operation and served as a bridge between SILS and CLS. However, only when the operator is proficient in CLS and has overcome the RPLS learning curve can the operator complete the operation with the help of a laparoscopic assistant, which not only saves manpower but also prevents intraoperative side injuries and improves operation time and efficiency. Although the multichannel puncture platform used in RPLS will impose some financial burden on patients, the short-term postoperative effect of patients suggests that it is a potentially feasible and inexpensive way to mitigate economic costs after surgery. Of course, we discovered a report that (25) can easily create this type of instrument platform during operation, which is simple to use, economical, and feasible. However, there are some concerns about this operation right now, such as a lack of training assistants and surgical teams. This operation, however, can only be performed after standardized and rational training and mastery of laparoscopic radical gastrectomy for gastric cancer. As a result, the operation continues to emphasize operation team cooperation and assistant training while placing greater emphasis on the operation's skill and safety. As a result, intraoperative side injury is avoided, perioperative complications are reduced, the operation is made less invasive, patients' postoperative rehabilitation is accelerated, and patients benefit. However, the RPLS umbilical incision length limitation is also one of the reference factors for tumour length and diameter selection. The resected tumour focus is bound to be removed from the umbilical single incision. If the tumour focus is too large, it may not be removed, so it is necessary to further extend the umbilical incision, which not only increases the trauma of RPLS but also prolongs the operation time and increases the probability of incision infection in the operation area. Therefore, the tumour length and diameter of all patients in this study were ≤4 cm to ensure the smooth removal of the tumour focus through the umbilical single incision.

## Conclusions

Our findings show that laparoscopic radical gastrectomy with reduced-port laparoscopy is clinically safe and feasible. Compared to CLS, it has the advantages of less trauma, fewer inflammatory reactions, and better cosmetic effects, which can accelerate patients' postoperative recovery and is more in line with the modern concept of rapid rehabilitation surgery and minimally invasive surgery.

Although the data from this study can be used to support clinical surgeons to perform this procedure, the sample size is small and based on a single-centre data study. More research samples are required to confirm the feasibility and safety of RPLS and to clarify the surgical application value of this method.

## Data Availability

The raw data supporting the conclusions of this article will be made available by the authors, without undue reservation.
