# Corrigendum: Comparison of clinical safety and feasibility between reduced-port laparoscopic radical gastrectomy and conventional laparoscopic radical gastrectomy: A retrospective study

**DOI:** 10.3389/fsurg.2022.1061238

**Published:** 2022-11-11

**Authors:** Liang Wang, Yingfang Deng, Su Yan, Xinfu Ma, Cheng Wang, Wei Miao, Xiaoqian Chen

**Affiliations:** ^1^ Gastrointestinal Oncology Surgery, Affiliated Hospital of Qinghai University & Affiliated Cancer Hospital of Qinghai University, Xining, China; ^2^ Medical-Oncology, Affiliated Hospital of Qinghai University & Affiliated Cancer Hospital of Qinghai University, Xining, China

**Keywords:** conventional laparoscopic surgery, reduced-port laparoscopic surgery, single-incision laparoscopic surgery, natural orifice specimen extraction surgery, gastric cancer

A corrigendum on Comparison of clinical safety and feasibility between reduced-port laparoscopic radical gastrectomy and conventional laparoscopic radical gastrectomy: A retrospective study By Wang L, Deng Y, Yan S, Ma X, Wang C, Miao W and Chen X. (2022) Front. Surg. 9: 995194. doi: 10.3389/fsurg.2022.995194

In the published article, there was an error in the author list, and author Yingfang Deng was excluded.

The corrected author list appears below:

Liang Wang^1†,^ Yingfang Deng^2†^, Su Yan^1^, Xinfu Ma^1^, Cheng Wang^1^, Wei Miao^1^ and Xiaoqian Chen^1*^

In the published article, there was an error with the author contributions. The correct author contributions appear below:

XC is the corresponding author. LW and YD are the first author. XC contributed to the study concept and design. SY and XM conducted the laparoscopic radical gastrectomy. LW and YD wrote the manuscript. CW and WM collected and analyzed the data. XC revised and edited the manuscript. SY and XM are the guarantors of this study. All authors contributed to the article and approved the submitted version.

The authors apologize for this error and state that this does not change the scientific conclusions of the article in any way. The original article has been updated.

